# Distinctly Different Dynamics and Kinetics of Two Steroid Receptors at the Same Response Elements in Living Cells

**DOI:** 10.1371/journal.pone.0105204

**Published:** 2014-08-18

**Authors:** Hatice Z. Nenseth, Xavier Dezitter, Martina Tesikova, Florian Mueller, Tove I. Klokk, Gordon L. Hager, Fahri Saatcioglu

**Affiliations:** 1 Department of Biosciences, University of Oslo, Oslo, Norway; 2 Computational Imaging and Modeling Unit, Institut Pasteur, Paris, France; 3 Laboratory of Receptor Biology and Gene Expression, National Cancer Institute, National Institute of Health, Bethesda, Maryland, United States of America; 4 Institute for Cancer Genetics and Informatics, Oslo University Hospital, Oslo, Norway; Institut de Génomique Fonctionnelle de Lyon, France

## Abstract

Closely related transcription factors (TFs) can bind to the same response elements (REs) with similar affinities and activate transcription. However, it is unknown whether transcription is similarly orchestrated by different TFs bound at the same RE. Here we have compared the recovery half time (t_1/2_), binding site occupancy and the resulting temporal changes in transcription upon binding of two closely related steroid receptors, the androgen and glucocorticoid receptors (AR and GR), to their common hormone REs (HREs). We show that there are significant differences at all of these levels between AR and GR at the MMTV HRE when activated by their ligands. These data show that two TFs bound at the same RE can have significantly different modes of action that can affect their responses to environmental cues.

## Introduction

The first and most critical step in regulation of gene expression is transcription which is a highly ordered process where protein complexes are sequentially recruited to target genes, including the specific transcription factors (TFs), the general TFs and RNA polymerases. Whereas the general TFs bind to well-characterized sites in the promoter, specific TFs bind to response elements (REs) that are either in the vicinity of or far away from the target genes. TFs bind to their REs with high precision which is the basis for the specificity of gene regulation in response to environmental cues that modulate TF activity.

It is known that related TFs can bind to and regulate transcription from the same RE. This may result in similar or opposing activities at the same RE, leading to corresponding transcriptional outcomes (for reviews, see [1]–[3]). However, the nature of the binding events, and whether the transcriptional program is similarly affected, is not known.

One group of TFs that can bind to and activate transcription from the same RE is the steroid receptors that belong to the nuclear receptor superfamily [4], [5]. Despite distinct roles of individual steroids, there are significant similarities in the REs recognized by their receptors. For example, the consensus hormone RE (HRE) for the glucocorticoid receptor (GR) is a family of related sequences composed of an imperfect palindrome of hexameric half sites separated by a 3-base pair spacer [6], [7] with some modifications identified recently in genome-wide analyses (e.g. [8]). This HRE is also recognized by the androgen receptor (AR), progesterone receptor (PR), and the mineralocorticoid receptor (MR) [9], [10]. These findings have raised the question as to how the selectivity of hormone action is achieved in cells where more than one steroid receptor is expressed and when their ligands are concurrently available.

There are several steps at which selective effects of two TFs that bind to the same RE can be achieved. First, recently documented rapid TF interaction with chromatin in living cells [11] could be different for the two TFs. Second, TFs may differentially and in a temporally distinct manner recruit cofactors and chromatin modifying complexes to the promoters they interact with (for reviews, see [12], [13]). Third, consequence of RE association of a TF on the local chromatin environment can vary for different TFs. To date, there is no thorough analysis of these different levels of regulation to determine whether different TFs can differentially affect them when bound at the same RE.

To compare the dynamics and activities of two closely related TFs at all of these different levels, we have used the prototypical and well characterized mouse mammary tumor virus (MMTV) promoter that contains HREs for steroid receptors. Using a cell system that has a tandem array of the MMTV promoter [14], we studied the fluorescence recovery half time (t_1/2_) measured by fluorescence recovery after photobleaching (FRAP), binding site occupancy determined by chromatin immunoprecipitation (ChIP), and transcription dynamics induced by AR compared with GR. We show that there are differences at all of these levels in response to agonist stimulation. These data indicate that two TFs of the same family bound at the same RE can have mechanistically different modes of regulating transcription which helps explain the selectivity in the activity of TFs with similar DNA binding specificities.

## Materials and Methods

### Chemicals

Methyltrienolone (R1881) was purchased from Dupont-NEN, and Dexamethasone (DEX) from Sigma. All chemicals were dissolved in 100% ethanol and used at working concentrations of 10 nM (R1881) and 100 nM (DEX). 5,6-dichloro-1-β-D-ribofuranosyl- benzimidazole (DRB) (Sigma) and actinomycin D (ActD) (Calbiochem) were dissolved in DMSO and used at working solutions of 100 µg/ml and 1 µM, respectively.

### Cell Culture

Stable cell lines expressing GFP-AR (3108) and GFP-GR (3617) under the control of the Tet-Off inducible system were previously described [14], [15]. The cells were grown at 37°C with 5% CO_2_ in Dulbecco's modified Eagle's medium (DMEM) supplemented with 10% fetal bovine serum, 2 mM L-glutamine, 5 mg/ml penicillin/streptomycin (Life Technologies, Inc.) and 10 µg/ml tetracycline (Sigma) (to suppress GFP-AR and GFP-GR expression). In preparation for experiments, cells were grown in this medium without tetracycline in order to induce expression of the receptor. Cells were then transferred to growth medium without tetracycline, containing 10% charcoal-stripped serum for 48 h to remove steroids. Prior to imaging experiments, cells were treated as described above, except phenol-red free medium was used to eliminate autofluorescence.

### Fluorescence Recovery After Photobleaching (FRAP)

3108 and 3617 cells were grown in MatTek plates for live cell imaging (Nunc) and treated with the agonists R1881 or DEX alone, or in combination with the transcription inhibitors DRB or ActD (time course of 0.5, 1.5, 2.5 and 4 h with R1881 or 1, 4 and 8 h with DEX, or for 1.5 h for transcription inhibition). FRAP analyses were carried out on an Olympus FluoView FV1000 confocal laser scanning microscope equipped with a PlanApo 60X 1.4 NA oil objective (Olympus, Hamburg, Germany) and a SIM scanner. Constant temperature was set to 37°C by an incubator enclosing the microscope stage. Five single prebleach images were acquired followed by a brief bleach pulse of 100 msec using 405-nm laser line at 100% laser power (laser output, 30%) without attenuation. Single optical sections were acquired at 500 msec intervals by using 488-nm laser line with laser power attenuated to 10%. Fluorescence intensities in the regions of interest were analyzed, and FRAP recovery curves were generated using Olympus FV10-ASW 1.7b software and Microsoft Excel as previously described [16]. All of the quantitative data for FRAP recovery kinetics were collected from 11–30 cells in total imaged on at least two separate days.

For semi-quantitative analysis and determination of t_1/2_, FRAP curve of each cell was interpolated to the same temporal sampling, and fit with a function which is the sum of three exponentials. From this fit, it was calculated how long it takes the curve to reach 50% of its final recovery value. For each experimental condition, each individual FRAP curve was fit with the model. After the extraction of the t_1/2_, the average t_1/2_ and corresponding standard error was calculated.

### Western Analysis

3108 and 3617 cells were left either untreated or treated with R1881 or DEX for 1, 4 and 8 h before the cells were harvested by scraping in PBS and collected by centrifugation. The cell pellet was washed twice in ice-cold PBS. Whole cell extract was prepared by resuspending the cells in 200 µl of lysis buffer (20 mM HEPES pH 7.4, 300 mM NaCl, 1.5 mM MgCl_2_, 0.2 mM EDTA, 0.1% Triton X-100, 0.5 mM DTT, 0.5 mM PMSF, protease inhibitor cocktail from Sigma). The suspension was rotated at 4°C for 1 h, followed by centrifugation at 13000 rpm for 10 min. The supernatant was collected after centrifugation, and the protein concentration was determined using the Bradford assay (BioRad). The proteins were resolved on a 7% SDS PAGE gel, and transferred to a PVDF membrane (BioRad). The membrane was blocked in 5% skimmed milk in Tris buffered saline (TBS)-0.1% Tween 20 and following by incubation with the primary antibodies for GFP (Invitrogen, 1∶1000) and α-tubulin (Sigma, 1∶4000) in 3% BSA in TBS-0.1% Tween 20. HRP-linked secondary antibodies (Sigma, 1∶5000–10000 in 0.5% skimmed milk in TBS-0.1% Tween 20) and an enhanced luminescence kit (SuperSignal West Dura Chemiluminescent Substrate, Thermo Scientific) were used for the detection of proteins. Images were obtained on a Kodak imaging station 4000R and the band intensities were determined using Carestream Imaging Software. The specific Western signals were quantified using Carestream Health imaging software. The measured intensity of GFP signals were then normalized to the corresponding α-tubulin signal. The Western blot images are representative of two independent experiments.

### Chromatin Immunoprecipitation Assay (ChIP)

ChIP experiments were carried out according to the standard protocol (Upstate Biotechnology) with some modifications. 3108 and 3617 cells were treated with R1881 or DEX alone, or in combination with the transcription inhibitors DRB or ActD (time course of 1, 2, 4 and 8 h with R1881 or 1, 4 and 9 h with DEX, or for 1.5 h for transcription inhibition), and crosslinked with 1% formaldehyde at 37°C for 10 min, followed by a quenching step with 125 mM glycine for 5 min. Cells were washed twice with ice-cold phosphate buffered saline (PBS) containing a complete protease inhibitor cocktail (PI; Roche Diagnostics), harvested in PBS plus PI and pelleted by centrifugation (2000 rpm, 5 min, 4°C). After lysis in ChIP lysis buffer (50 mM Tris-HCl pH 8, 10 mM EDTA, 0.5% SDS, PI) for 1.5 h on ice, chromatin was sonicated at high intensity for 30 min (3x 10 bursts of 30 sec ON and 30 sec OFF; 4°C) using the Bioruptor sonicator (Diogenode). After centrifugation (13000 rpm, 10 min, 4°C), sheared chromatin was diluted in ChIP dilution buffer (0.01% SDS, 1% Triton X-100, 1.2 mM EDTA, 16.7 mM Tris-HCl pH 8.1, 167 mM NaCl, PI) to 25 µg/ml, precleared with 50% slurry of protein A-agarose beads (Millipore) (2 h, 4°C), and immunoprecipitated overnight with an anti-GFP (Invitrogen), anti-elongating RNA polymerase II (Pol II phosphoS2; Abcam) or non-specific rabbit IgG (Vector Laboratories) antibody at 4°C on a rotating platform. Antibody-bound chromatin complexes were incubated with 60 µl of protein A-agarose beads for 1.5 h at 4°C. Antibody-chromatin-bead complexes were then washed with each ChIP wash buffer plus PI once for 15 min in the following order: Low salt immune complex buffer (0.1% SDS, 1% Triton X-100, 2 mM EDTA, 20 mM Tris-HCl pH 8.1, 150 mM NaCl), high salt immune complex buffer (0.1% SDS, 1% Triton x-100, 2 mM EDTA, 20 mM Tris-HCl pH 8.1, 500 mM NaCl) LiCl immune complex buffer (0.25 M LiCl, 1% IGEPAL-360, 1% sodium deoxycholate, 1 mM EDTA, 10 mM Tris-HCl pH 8.1) and TE (10 mM Tris-HCl pH 8, 1 mM EDTA), and antibody-chromatin complexes were eluted twice in SDS buffer (first in 1% SDS, 0.1 M sodium bicarbonate; second in 1.5% SDS, 0.1 M sodium bicarbonate). Formaldehyde crosslinking was reversed in elution buffer (containing 40 mM Tris-HCl pH 6.5, 10 mM EDTA, 200 mM NaCl, proteinase K from Roche Diagnostics) at 65°C overnight, followed by DNA purification using phenol-chloroform-isoamyl alcohol mixture (Sigma-Aldrich) and ethanol precipitation. Immunoprecipitated DNA, as well as input DNA, was eluted in 50 µl of water and quantified by quantitative polymerase chain reaction (qPCR) on a LightCycler 480 instrument (Roche Diagnostics) with the LightCycler 480 SYBR Green I Master Mix (Roche Diagnostics) in duplicates using primer sets specific for the MMTV-LTR Nuc-B region. Primer sequences are available upon request. ChIP assay was performed as duplicates of two independent experiments.

### Quantitative Reverse Transcription-PCR (qRT-PCR)

3108 and 3617 cells were grown in triplicates on 6-well dishes for 48 h, and left either untreated (CTR) or treated with R1881/DEX, or the inhibitors DRB/ActD alone or in combination for 1.5 h (agents were added at the same time as ligands). Total RNA was extracted with Trizol reagent (Invitrogen) according to manufacturer's recommendations, followed by elution using QIAGEN RNeasy columns. RNA was then subjected to first-strand cDNA synthesis using the SuperScript II system (Invitrogen). *V-ras* expression levels were determined by qPCR in duplicates using the LightCycler 480 instrument (Roche) with the LightCycler 480 SYBR Green I Master Mix (Roche). A standard curve was created by serial dilutions of cDNA to calculate the relative amount of *Ras* and *Rplp0* for each sample. These values were then normalized to the relative amount of *Rplp0*. qRT-PCR results were obtained from two independent experiments.

## Results

### Androgen and glucocorticoid receptors have distinct dynamics and transcription kinetics at the same HRE

We have previously generated a cell line to study *in vivo* dynamics of AR with chromatin, 3108 cells [15], based on the model system developed for GR, 3617 cells [14]. These two mouse mammary adenocarcinoma cell lines were generated by stable transfection of a green fluorescent protein (GFP) tagged AR (pTRE-Tight-GFP-AR) or GR (pTet-nGFP-C656G) construct under tetracycline repressible promoter into the same parental cell line (3134 cells). 3134 cells contain approximately 200 copies of a 1.3 kb MMTV-LTR sequence fused to a 600 bp fragment of Harvey sarcoma virus encoding the *v*-*Ras* gene product as a reporter integrated into chromosome 4 in a tandem fashion; this enables the direct visualization of GFP tagged AR or GR binding to the MMTV promoter in live cells. As each copy harbors binding sites for four to six receptor molecules, the MMTV array has a capacity for about 1000 receptor molecules. In the genome, the MMTV promoter is characterized by a series of six positioned nucleosomes (A-F); ligand bound AR and GR can bind to the HREs located in the nucleosome B/C region, promoting transcription of the Ras reporter gene [17], [18]. Using these cells and FRAP allows the measurement of highly dynamic interactions between the receptor and the DNA template in living cells [19]. GFP-AR and GFP-GR bind to the same HREs in the MMTV LTR and the hormone response of promoters within the MMTV array is indistinguishable from that of a single-copy gene [20], [21].

**Figure 1:**
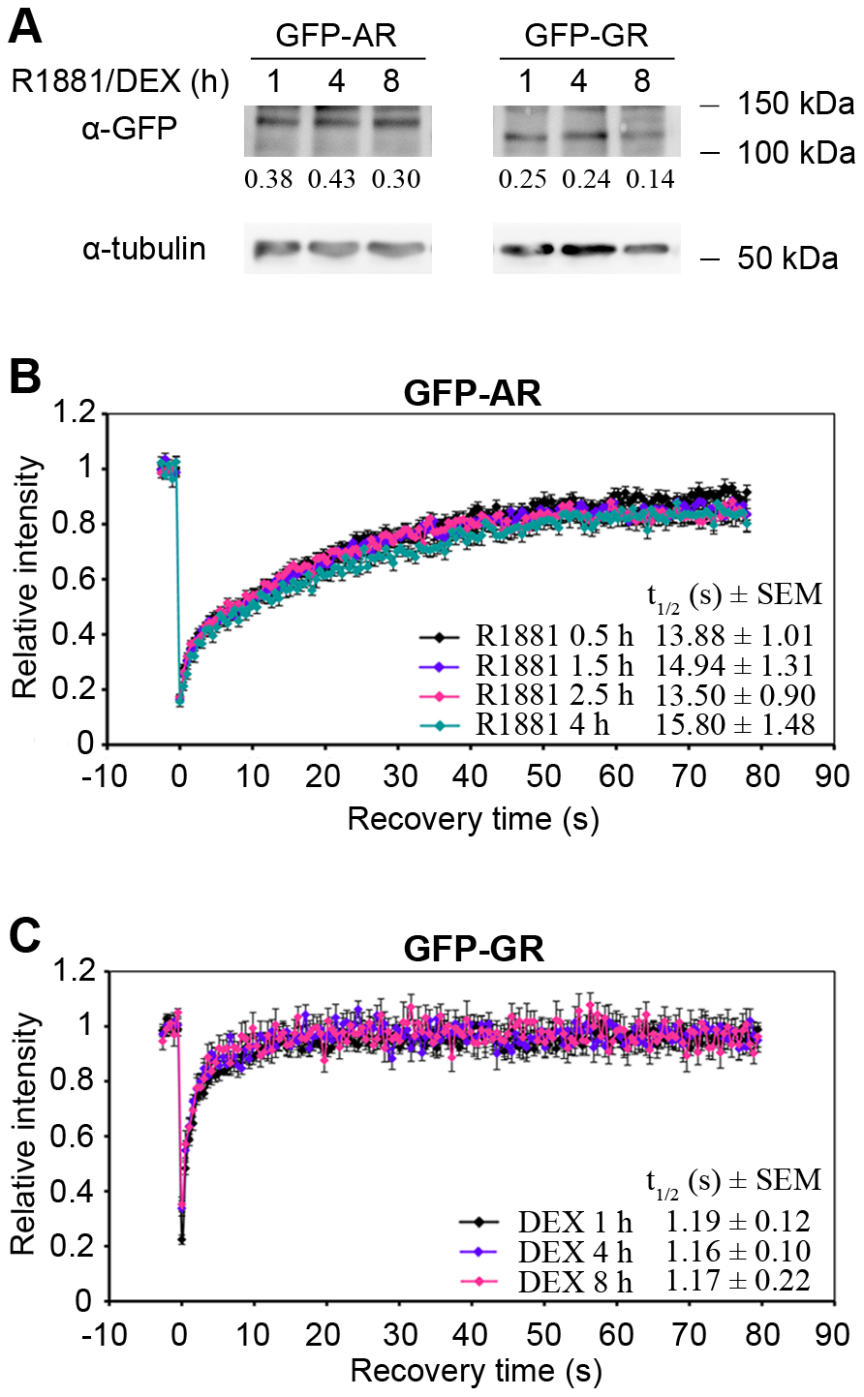
Distinct mobilities of GFP-AR and GFP-GR at the MMTV array in living cells. (**A**) GFP-AR and GFP-GR expression in 3108 and 3617 cells, respectively. Cells were treated with agonists R1881 or DEX as indicated. Total cell extracts were subjected to Western analysis using an anti-GFP antibody (upper panels) and an anti-α-tubulin antibody as loading control (lower panels). Relative quantification of band intensities is indicated below the lanes; α-tubulin at each time point was set to 1.0. (**B**, **C**) GFP-AR and GFP-GR show distinct FRAP recovery dynamics at the MMTV array. 3108 cells (**B**) and 3617 cells (**C**) were treated with agonists R1881 or DEX for the given time periods. GFP fluorescence at the MMTV array was bleached and recovery after bleaching was followed by live cell imaging. FRAP curves were generated, and recovery half times were determined in a semi-quantitiave manner. Error bars represent means ± standard error.

Previous experiments determined the recovery half time of GFP-GR and GFP-AR in FRAP experiments where the region of MMTV array appeared as a bright spot in the nucleus was selectively photobleached and then the time at which fluorescence enters the bleached region was measured. The fluorescence recovery contains information about the diffusion rate of GFP-AR and GFP-GR plus any binding interactions with large, relatively immobile substrates [14], [15], [19]. However, these were done at single time points and in different conditions for GFP-AR and GFP-GR. To assess whether there are any changes to the recovery half-time of the receptors during the course of the transcription response, FRAP analysis was carried out at different time points after hormone addition. As shown in Figure 1A, GFP-AR expression increased slightly by 4 h after hormone addition and decreased by 8 h, whereas GFP-GR expression was similar at 1 and 4 h which decreased by 8 h. The FRAP recovery curves for GFP-AR or GFP-GR were similar at different time points after hormone addition (Figures 1B and 1C). Interestingly, under these experimental conditions and using the same equipment and settings, the recovery half-time for GFP-AR was significantly slower compared with that for GFP-GR (compare Figure 1B and 1C). This indicates that GFP-AR interactions with the MMTV HREs are stronger compared with GFP-GR.

**Figure 2:**
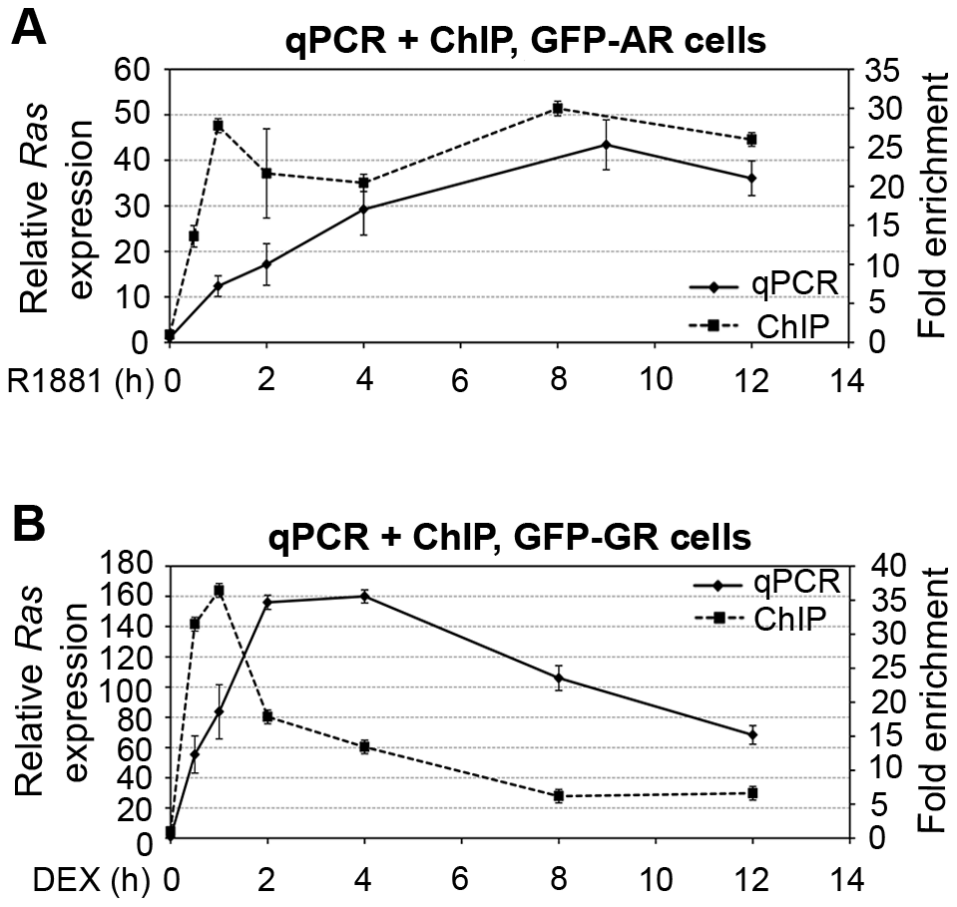
GFP-AR and GFP-GR differentially associate with and activate transcription from the MMTV LTR. Time course of GFP-AR (**A**) and GFP-GR (**B**) transcriptional activity and promoter occupancy at the MMTV LTR. 3108 cells (AR) in (**A**) or 3617 cells (GR) in (**B**) were treated with agonists R1881 or DEX for the given time points. MMTV-Ras transcript levels were examined by qPCR (y-axis on the left-hand side). ChIP analysis was performed using an anti-GFP antibody, and receptor occupancy at MMTV LTR was determined by qPCR (y-axis on the right-hand side). Error bars represent means ± standard deviation.

To determine the kinetics of GFP-AR-mediated transcription at the MMTV LTR in 3108 cells, we performed a time-course experiment and determined reporter gene *Ras* expression by qPCR. As shown in Figure 2A, *Ras* mRNA levels continued to increase upon R1881 addition reaching maximal levels at around 8 h which did not significantly change thereafter up to 12 h. The *Ras* expression profile in response to R1881 was qualitatively similar by RNA FISH (see Figure S1 and Method S1).

We next determined GFP-AR occupancy levels at the MMTV promoter during the same time course using chromatin immunoprecipitation (ChIP) which gives an average of bound molecules in the whole cell population during the time it takes to fix the interactions. There was a rapid increase in GFP-AR occupancy at the MMTV HRE upon hormone activation reaching maximal levels of ∼25–30-fold higher than basal levels by 60 min and remained essentially unchanged through the course of the experiment up to 12 h indicating that GFP-AR mediated transcription profile is similar to the GFP-AR occupancy at the MMTV array (Figure 2A).

We then carried out similar experiments with GFP-GR in 3617 cells. As shown in Figure 2B, GFP-GR activation of MMTV-*Ras* was significantly greater and more rapid compared with GFP-AR reaching maximum levels by 2 h and declining rapidly thereafter, consistent with previous findings [22]. ChIP analysis showed that in parallel with *Ras* expression, GFP-GR occupancy at the MMTV HRE increased ∼35-fold by 1 h and then rapidly declined to 50% at 2 h and to 20% at 8 h (Figure 2B). Overall, GFP-GR mediated transcription profile followed receptor binding profile at the MMTV array.

### Transcription inhibitors differentially affect GFP-AR and GFP-GR mobility coupled to Pol II association

**Figure 3:**
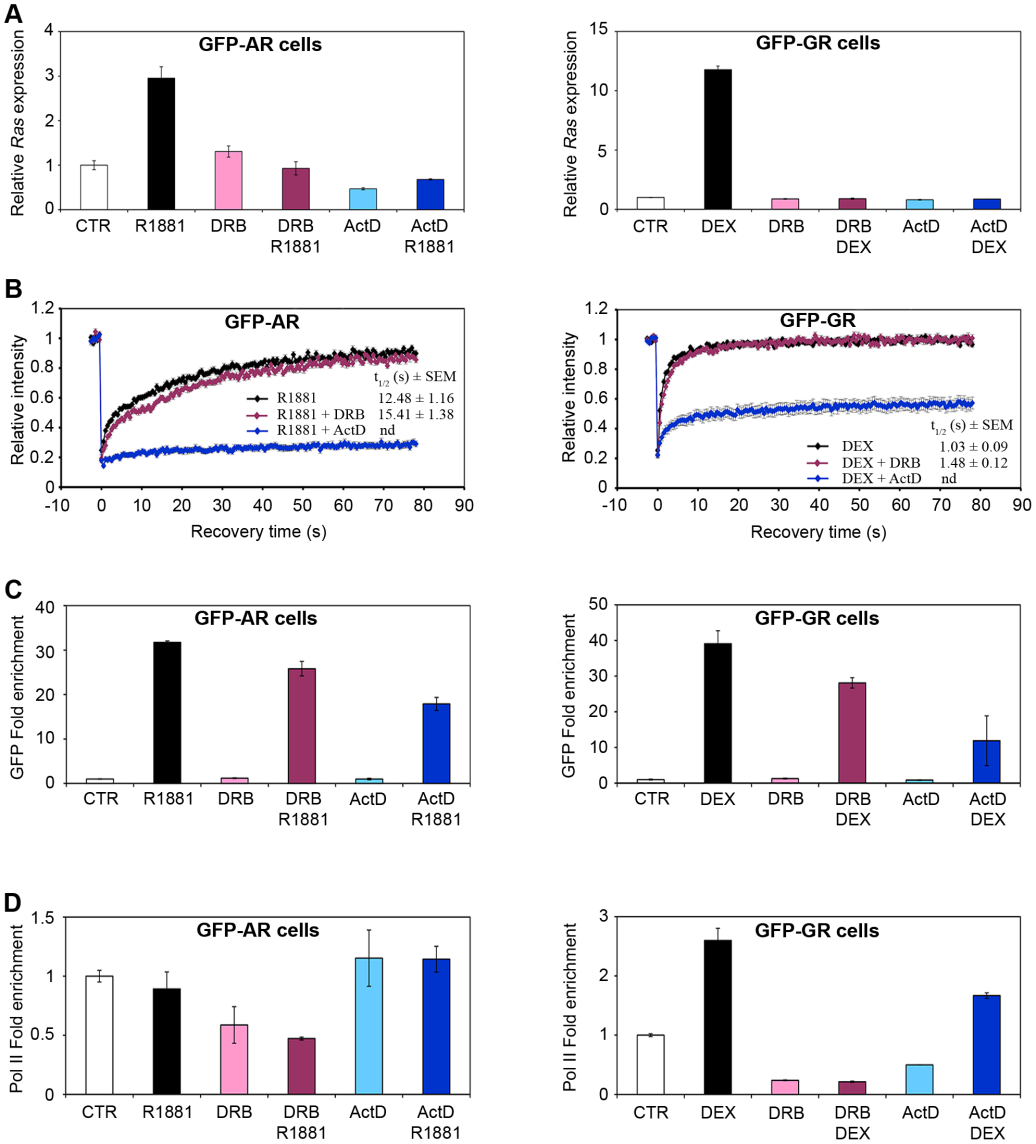
Transcription complexes at the MMTV promoter are differentially sensitive to RNA Pol II inhibitors. (**A**) DRB and ActD inhibit hormone induced Ras transcription in 3108 or 3617 cells. (**B**) DRB and ActD have differential effects on GFP-AR and GFP-GR mobility at MMTV LTR. After treatment with agonist R1881 or DEX alone or in combination with inhibitors DRB or ActD, GFP-AR (3108 cells) and GFP-GR (3617 cells) bound to the MMTV-LTR array were subjected to semi-quantitative FRAP analysis. The recovery half time values for ActD treatment were not determined (nd) as the fluorescence recovery was not large enough during the experimental time period. Error bars represent means ± standard error. (**C**) DRB and ActD decrease GFP-AR and GFP-GR occupancy at the MMTV array. 3108 or 3617 cells were treated as in (**A**). qPCR was used to validate ChIP analysis performed with an anti-GFP antibody using known binding sites at MMTV LTR. Error bars represent means ± standard deviation. (**D**) DRB and ActD have distinct effects on elongating Pol II at the MMTV array. 3108 or 3617 cells were treated as indicated in (**A**). qPCR was used to validate ChIP analysis performed with an anti-Pol II-pSer2 antibody using known binding sites at MMTV promoter. Error bars represent means ± standard deviation.

In order to determine whether the differential activities of GFP-AR and GFP-GR at the MMTV array are linked to transcription, we used two Pol II inhibitors, 5,6-dichloro-1-β-D-ribofuranosylbenzimidazole (DRB) and actinomycin D (ActD). DRB, a protein kinase inhibitor, prevents Pol II phosphorylation on Serine 2 and productive elongation [23], [24] whereas ActD intercalates to DNA and blocks Pol II progression [25]. As shown in Figure 3A, both agents efficiently inhibited hormone-induced transcription by both GFP-AR and GFP-GR, as expected.

We then checked whether transcription inhibition by these agents affects GFP-AR and GFP-GR mobility using FRAP analysis. We found that whereas DRB treatment did not have an effect, ActD led to a temporarily immobilized fraction for either receptor (Figure 3B). These data show that DRB and ActD differentially affect mobility of GFP-AR and GFP-GR in the nucleus.

Next we checked GFP-AR and GFP-GR occupancy at the MMTV LTR by ChIP analysis in response to DRB and ActD. As shown in Figure 3C, occupancy levels of both receptors at the MMTV HREs were decreased by 20% and 30% in the presence of DRB, whereas this decrease reached 40% and 70% with ActD for GFP-AR and GFP-GR, respectively.

As both DRB and ActD block transcription by inhibiting elongating Pol II, we examined Pol II-pSer2 levels at the MMTV array after DRB and ActD treatment by ChIP. As expected, in the presence of DRB, Pol II-pSer2 loading was decreased in both cell lines, with a stronger effect in GFP-GR cells (Figure 3D). Interestingly, ActD significantly decreased Pol II-pSer2 levels in response to GFP-GR activation, but not for GFP-AR.

Altogether, these data show that there are important differences in the way the two receptors associate with HREs. Furthermore, these results indicate that the transcription complexes that are assembled are differentially sensitive to DRB and ActD with distinct consequences for the transcriptional output.

## Discussion

The binding of a TF to its target RE is central to all aspects of development and homeostasis in metazoans as well as in lower organisms. TFs most often belong to families whose members are typically generated by duplication events during evolution and thus have closely related DNA binding domains and structures. For example, in metazoans TF families include bHLH, Mef2, Fox, Sox, ETS, Rel/Nf-kB, bZIP, Smad, and nuclear receptor proteins that have many members and even more closely related subgroups within each family. Whereas different mechanisms, such as divergence in the DNA binding domain sequences and ability to differentially heterodimerize with distinct partners, can generate TF specificity, some members of a TF family often bind and regulate transcription from the same REs interacting with the same cofactors. It has thus been unknown as to whether different, but related TFs would use similar mechanisms and activate transcription in the same manner when bound at the same RE. Here we have used two members of the nuclear receptor family of TFs, AR and GR, and a common HRE that they bind to and activate transcription from, to explore this basic question.

The first level of comparison we undertook was the mobility of receptors. Due to the availability of the unique cell system with an integrated array of the natural MMTV promoter containing multiple HREs, it is possible to observe binding events by steroid receptors in living cells and study their dynamics [14], [15], [26]. Since in previous studies the cell culturing conditions, length of treatments, as well as the equipment for FRAP analysis were different which can all affect the calculated recovery half times, we compared the dynamics of GFP-AR and GFP-GR under the same conditions using the same equipment. As shown in Figure 1B-C, we found that GFP-GR had a faster fluorescence recovery curve (i.e. short recovery half time) compared with that of GFP-AR. Because GFP-AR and GFP-GR are similar in size, expression level (Figure 1A), primary and tertiary structure of the DNA binding domain [27], [28], and their affinity towards the MMTV HREs [29], [30], the basis of this difference is not clear at present. One possibility is that the two receptors bind different cofactors that could affect the FRAP curve due to their binding affinity or cooperative effects of whole complexes; however, there is no known clear distinction in the cofactors that GR and AR interact with when bound to the MMTV HRE. Another possibility is that GFP-AR and GFP-GR binding to the HREs differentially affects local chromatin structure or epigenetic marks which in turn could alter recovery half times. Examination of global changes in acetylation as well as some specific histone marks has not indicated any differences in this regard (data not shown). A third possibility is that during the activation process the receptors themselves could differentially be modified posttranslationally which could affect their function and/or binding kinetics. For example, AR has been shown to be modified by phosphorylation, sumoylation, acetylation, or methylation events [31]–[34] at least some of which have not been described for GR. Another possibility is differential involvement of chaperone proteins, such as HSP90, or the proteasome, since both of these are implicated in the regulation of AR or GR dynamics and activity [19], [35]–[37]. Further work is needed to assess these possibilities.

Previous studies have shown that the kinetics of steroid receptor action at different REs can be quite complex due to alternate activation and repression phases. For example, microarray profiling revealed at least 12 distinct modes of action for GR at different HREs [38]. Thus, we asked whether GFP-AR and GFP-GR have differences in the kinetics of transcription at the MMTV HREs. GFP-AR mediated transcription increased gradually reaching maximal levels by 8 h and was stable at least up to 12 h (Figure 2). In contrast, and consistent with previous work (e.g. [39]), GFP-GR activated transcription rapidly, reaching maximal levels by 2 h upon which levels declined rapidly. The contrasting kinetic profiles suggest that the mode of action of the two receptors at the MMTV HRE is different and that they would give rise to distinct responses at this gene locus upon activation. At present the precise mechanism underlying the differential dynamic behaviour of the GFP-AR and GFP-GR at the MMTV promoter is not known. The differences could derive from modification status of the receptors or the promoter, the composition of the associated coactivator/corepressor complexes, as well as promoter structure and MMTV array size during the time-course of transcriptional regulation.

To follow the promoter occupancy of GFP-AR and GFP-GR during transcription activation at the MMTV array, we performed ChIP analysis. The current model suggests that the occupancy and residence times of a TF at a RE are not strictly correlated and can be measured independently. Interestingly, studies with the yeast TF, Rap1 indicates TF dynamics correlates more strongly than occupancy with genomic function [40]. Thus, given that GFP-AR has a slower FRAP curve (i.e. longer recovery half time) compared with GFP-GR, the prediction was that GFP-AR ChIP signals at the MMTV HRE should be higher than that for GFP-GR when both receptors were activated. However, this was not the case; in fact, GFP-GR ChIP signals, at the time at which FRAP measurements were taken, were similar to that observed with GFP-AR (Figure 2). This suggests that, at least in some cases, there may be no direct correlation between the TF kinetics and occupancy at REs.

There are several possibilities that can explain these results. One possibility is that the frequency of interactions by GFP-GR at the HRE among the whole cell population is higher compared with GFP-AR and thus this can ‘compensate’ for faster fluorescence recovery kinetics. This is possible since we did not observe any changes in the fluorescence recovery curves at different times after receptor activation when the transcription output was variable (Figure 1B-C and Figure 2). In spite of the similar magnitude of the ChIP signals at the time points with highest transcription, there was a significant difference in the time course of GFP-AR and GFP-GR association with the MMTV: whereas GFP-AR gradually reached its steady state loading levels by 1 h and did not significantly change after that, GFP-GR also reached highest levels at 1 h, but rapidly declined thereafter decreasing back to almost basal levels by 12 h (Figure 2). At present we do not know the reason for these differences. As in the recovery half time difference, these observations could be due to differential posttranslational changes to the receptor, its associated cofactors, or the chromatin template.

To determine whether binding events of GFP-AR and GFP-GR at the MMTV may require active transcription, we used transcription inhibitors, DRB and ActD. Interestingly, although not interfering with mobility of receptors, DRB significantly decreased the levels of receptors bound to MMTV HREs. In contrast, ActD significantly slowed down both GFP-AR and GFP-GR FRAP curves and diminished occupancy (Figure 3B, 3C). Recently, ActD was suggested to inhibit trafficking of GR to the nucleus in mouse thymocytes [41]. However, microscopy experiments did not show the same effect of ActD on GFP-AR and GFP-GR. These data indicate that the gene regulation by the promoter-associated GFP-AR and GFP-GR interacting with cofactors and Pol II is regulated in a complex manner. The observed differential dynamics of GFP-AR and GFP-GR at the MMTV promoter during the time-course of transcriptional activation may impact the transcription initiation complex. For example, it is possible that promoter occupancy of GFP-AR and GFP-GR may be linked to the transcription elongation process. Indeed, it has been reported that positive transcription elongation factor b (P-TEFb) interacts with AR and enhances efficiency of transcription elongation [42]. In addition, large inactive P-TEFb complex has been shown to be disrupted by ActD that was also shown to inhibit interactions between TF and its binding site [43], [44]. Thus, the receptor association with HREs may be directly related to elongation events.

The discrepancy between DRB and ActD effects on receptor mobility and occupancy levels may also be explained by the differential action of inhibitors on transcription elongation. DRB is an inhibitor of Pol II-Ser2 phosphorylation and may *per se* not interfere with receptor binding dynamics. In contrast, as ActD intercalates to DNA, it might change the local chromatin structure around the HRE and affect receptor-chromatin interactions. Considering that ChIP analysis determines occupancy levels, ActD may interfere with frequency of receptor-chromatin interactions as a whole in the cell population without affecting receptor mobility in single cells.

There was a decrease in Pol II-pSer2 levels at the MMTV array in the presence of DRB in both GFP-AR and GFP-GR cells, as expected. Interestingly, we have observed that Pol II is differentially recruited to the MMTV array during a time course of transcription activation by GFP-AR and GFP-GR (data not shown). This suggests that in case of activation by GFP-AR Pol II might be poised at the transcription start site. On the other hand, ActD decreased Pol II-pSer2 levels only in the presence of GFP-GR. Since ActD was shown to promote accumulation of phosphorylated Pol II-Ser2 [45], it may explain the binding of Pol II-pSer2 in the presence of GFP-AR. However, it is clear that ActD affects transcription at different levels with a decrease in transcriptional outcome. These data suggest that the ‘communication’ between the two receptors and the transcription machinery may be differentially achieved.

The precise molecular basis for the striking differences between GFP-AR and GFP-GR activity bound at the same HRE are currently not clear. Previous studies have suggested that amino acid differences in the steroid receptors might contribute to altered transcriptional outcomes at the same HRE [29], [46], [47]; however, most of this previous work was conducted using reporter plasmids and transient transfection assays and did not provide any mechanistic explanation. It has also been reported that binding to non-conventional HRE sequences might lead to differences in receptor activity, or that non-receptor factors are involved in further regulating specificity of steroid receptor functions acting at the same HRE [48], [49]. Regardless of the mechanism, the data we have presented suggest that the dynamics and presentation of the two receptors bound at the HREs to the transcriptional initiation machinery and the RNA Pol II complex are distinctly different which may affect the FRAP curves and promoter binding kinetics; this, in turn, is likely to engage the basal transcription apparatus differently in response to extracellular stimuli. Thus, even though every gene may have a set transcription regulation program, this can be orchestrated differently by related but distinct TFs bound at the same REs through distinct mechanisms.

## Supporting Information

Figure S1 **GFP-AR induced transcription from the MMTV array.** 3108 cells (GFP-AR) were left either untreated or treated with R1881 for the given time points. RNA FISH analysis was performed on fixed cells. The FISH signals were detected by confocal microscopy and quantified from >110 randomly chosen cells for each time point. Error bars represent means ± standard error.
(TIF)

Method S1 **Supplemental Method including Supplemental Literature.**
(DOC)
