# Supplementary figures and images for: Distinctly Different Dynamics and Kinetics of Two Steroid Receptors at the Same Response Elements in Living Cells
Source: PLoS One. 2014 Aug 18;9(8):e105204. doi: 10.1371/journal.pone.0105204 (PMC4136857; doi:10.1371/journal.pone.0105204)

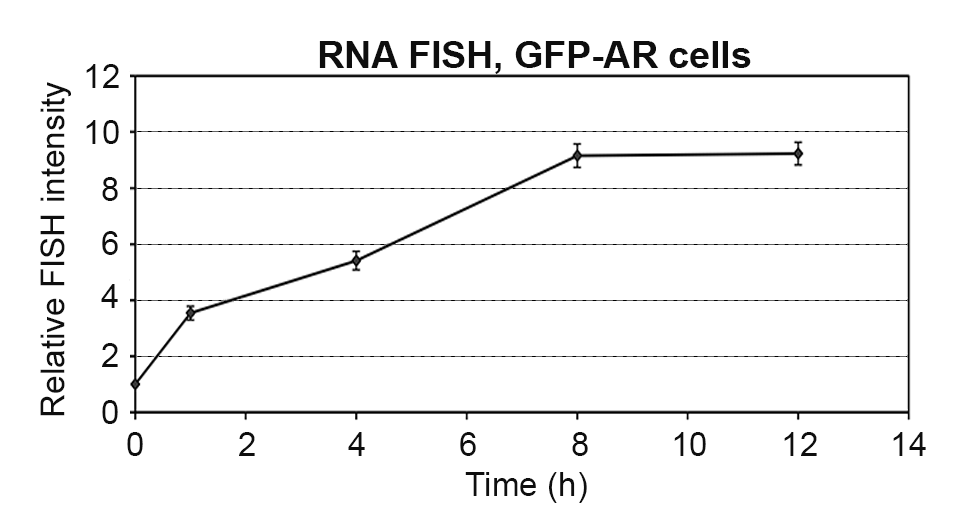

Supplement: Figure S1 — GFP-AR induced transcription from the MMTV array. 3108 cells (GFP-AR) were left either untreated or treated with R1881 for the given time points. RNA FISH analysis was performed on fixed cells. The FISH signals were detected by confocal microscopy and quantified from >110 randomly chosen cells for each time point. Error bars represent means ± standard error. (TIF) [file pone.0105204.s001.tif]
