# Supplementary material for: Distinctly Different Dynamics and Kinetics of Two Steroid Receptors at the Same Response Elements in Living Cells
Source: PLoS One. 2014 Aug 18;9(8):e105204. doi: 10.1371/journal.pone.0105204 (PMC4136857; doi:10.1371/journal.pone.0105204)
Supplement: Method S1 — Supplemental Method including Supplemental Literature. (DOC) [file pone.0105204.s002.doc]

**Method S1**

*RNA Fluorescence In Situ Hybridization (RNA FISH):* The cells were grown on cover slips placed in six-well culture plates. Prior to the experiment cells were left either untreated or treated with R1881 for the given time points. Cells were then fixed with 3% paraformaldehyde and processed for RNA FISH to detect the MMTV-Ras expression as described previously . Images were acquired on an Olympus FV1000 upright confocal laser scanning microscope with a PlanApo 60X 1.4 NA oil immersion objective. The FISH signals were quantified using Olympus FV10-ASW 1.7b software after subtraction of the background nuclear fluorescence. The integrated total RNA FISH intensity was calculated for each condition and normalized to the levels in untreated cells to obtain relative RNA FISH intensity. Representative images were processed with ImageJ.

**Supporting literature:**

1. Muller WG, Walker D, Hager GL, McNally JG (2001) Large-scale chromatin decondensation and recondensation regulated by transcription from a natural promoter. J Cell Biol 154: 33-48.
